# Longitudinal association between chronic diseases and fall risk among middle-aged and older adults in China: the mediating role of activity limitations

**DOI:** 10.7189/jogh.16.04106

**Published:** 2026-03-13

**Authors:** Derong Huang, Yingjie Fu, Wen Yang, Biying Xu, Zhengfei Yang

**Affiliations:** 1 Quality Assessment Center, Sun Yat-sen Memorial Hospital, Sun Yat-sen University, Guangzhou, China; 2 Department of Critical Care Medicine, Sun Yat-sen Memorial Hospital, Sun Yat-sen University, Guangzhou, China; 3 Affiliated Hospital of Shandong University of Traditional Chinese Medicine, Jinan, China

## Abstract

**Background:**

Falls are a major public health issue for middle-aged and older adults, especially those with chronic diseases. While a correlation is known, the longitudinal relationship and the underlying mechanisms between chronic diseases and falls remain unclear. This study investigates the longitudinal association and examines the mediating role of activity limitations.

**Methods:**

A total of 2400 middle-aged and older adults who completed all three waves (2013, 2015, and 2018) of the China Health and Retirement Longitudinal Study (CHARLS) were included. Mixed-effects logit models with time-fixed effects were employed to examine the longitudinal association between chronic diseases and fall risk. The association pattern between the number of chronic diseases and the probability of falling was assessed by incorporating quadratic terms, constructing restricted cubic splines, and plotting dose-response relationships. Mediation analysis was conducted to evaluate the role of activity limitations as a mediator.

**Results:**

The incidence of falls and multimorbidity increased over the study period. The number of chronic diseases was significantly associated with a higher fall risk (β = 0.138, *P* < 0.01), exhibiting a linear dose-response relationship. Mediation analysis based on the disablement process model indicated that activity limitations accounted for 21.08% of this association.

**Conclusions:**

The risk of falls among Chinese middle-aged and older adults increases linearly with the number of chronic diseases, an association partly mediated by activity limitations. Fall prevention strategies should prioritise managing activity limitations through targeted assessments and interventions, such as physical exercise, to mitigate fall risk in this population.

The World Health Organization identifies falls as the second leading cause of accidental injury deaths worldwide, following road traffic injuries [1]. As such, falls have emerged as a critical global public health issue [2], posing serious threats to the physical and mental well-being of middle-aged and older adults. The problem is particularly pronounced in China, where evidence suggests a fall incidence of 18–19% in this population [3]. Falls can lead to severe adverse outcomes, including fractures [3], injuries [4], social isolation [5], depression [6], disability [3], and even death [7]. Beyond being a leading cause of injury, falls contribute significantly to disability-adjusted life years (DALYs) lost among the older population, imposing a substantial economic burden on health care systems globally [8]. In China, with its rapidly ageing demographic, the economic and social costs associated with fall-related injuries are projected to increase dramatically [9]. Given the profound implications of falls, which extend to direct health outcomes and to psychological and social functioning, enhancing prevention and intervention strategies is a public health priority.

Older adults with chronic diseases are at especially high risk of falls [10]. While numerous studies have established a statistically significant association between the number of chronic conditions and fall risk [11], indicating that the probability of falling increases with disease accumulation, the longitudinal pattern of this relationship remains unclear. Some studies have utilised two-wave data to explore this association [12], extending beyond cross-sectional analyses. However, such designs cannot establish temporal precedence between exposure and outcome, limiting causal inference and the ability to capture dynamic longitudinal associations.

The mechanisms through which chronic diseases influence fall risk are also not well understood, hindering the development of effective interventions. The disablement process model [13], a seminal framework in gerontology, posits a pathway from chronic conditions (pathology) to impairments in specific physiological systems, which subsequently lead to functional limitations in daily activities and ultimately result in disability. Grounded in this conceptual model, the present study operationalises chronic disease burden as the initial pathology, activity limitations as the mediator, and fall incidence as a critical disabling outcome. Chronic conditions often signal declining physical function [14], and falls are closely related to impairments in gait, balance, and muscle strength [15,16]. Thus, chronic diseases may elevate fall risk by exacerbating activity limitations. Although previous studies have separately linked activity limitations to both chronic disease burden and fall risk, no longitudinal research has concurrently examined activity limitations as a mediator in this relationship. Therefore, this study aims to use multi-wave longitudinal data to determine whether the number of chronic diseases is associated with falls, elucidate the pattern of this association, and specifically test the mediating role of activity limitations.

## METHODS

### Study design

**Figure 1:**
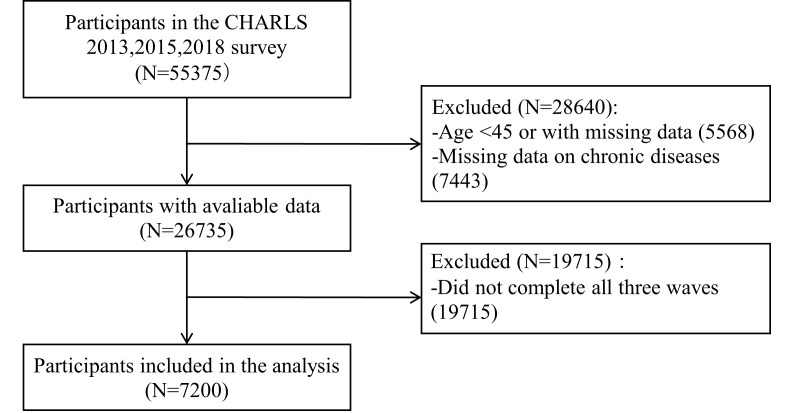
Flowchart of the selection of the study population from the CHARLS.

Data were obtained from the China Health and Retirement Longitudinal Study (CHARLS), conducted by Peking University. The baseline survey was launched in 2011, with five rounds of data collection completed by 2020. Using probability proportional to size sampling, CHARLS covers a nationally representative sample of adults aged 45 and older from 150 counties and 450 communities across 28 provinces. The survey collects extensive information on demographics, health status, health service utilisation, and medical insurance. To examine longitudinal associations, data from the 2013, 2015, and 2018 waves were merged into a panel data set. This study excluded individuals who were under 45 years of age, had missing data for any core variable (including the number of chronic diseases, fall history, and activity limitations), or did not complete all three survey waves (**Figure 1**). Consequently, a total of 2400 individuals were included in the final analytical sample. This resulted in a total of 7200 observations for the longitudinal analysis. Our analysis aligns with the Journal of Global Health’s Guidelines for Reporting Analyses of Big Data Repositories Open to the Public (Table S1 in the **Online Supplementary Document**).

### Measures

#### Measurement of chronic diseases

Chronic diseases were assessed based on the question: ‘Has a doctor ever told you that you have any of the following chronic conditions?’ The list encompassed 14 specific conditions: hypertension, dyslipidaemia, diabetes or elevated blood glucose, cancer or any malignant tumor, chronic lung diseases (such as chronic bronchitis, emphysema, or cor pulmonale), liver disease, heart disease, stroke, kidney disease, stomach or other digestive system diseases, emotional or psychiatric problems, memory-related disease, arthritis or rheumatism, and asthma. Initially, chronic diseases were treated as a categorical variable: no chronic disease, one chronic disease, and multimorbidity (≥2 chronic diseases), consistent with existing literature [17]. For correlation and mediation analysis, the number of chronic diseases was treated as a continuous variable. To prevent extreme values from undermining the robustness of the results, such as distorting the dose-response relationship in the main study population or exerting undue influence on model parameters, and in accordance with established research practice [15,18], individuals with six or more chronic diseases were combined into a ‘6+’ category (Table S2 in the **Online Supplementary Document**).

#### Measurement of fall

A fall was defined by the World Health Organization (WHO) as ‘an event which results in a person coming to rest inadvertently on the ground or floor or other lower level’ [1]. Falls were ascertained by asking participants if they had fallen since the last interview and were coded as a dichotomous variable (1 = yes, 0 = no).

#### Measurement of activity limitations

Activity limitations were measured as a continuous variable using combined scores from the Activities of Daily Living (ADL) and Instrumental Activities of Daily Living (IADL) scales. The ADL scale includes six items (dressing, bathing, eating, getting in/out of bed, toileting, and controlling urination/defecation), and the IADL scale includes five items (doing household chores, preparing meals, shopping, managing finances, and taking medications). Each item was scored from 0 (‘No, I do not have any difficulty’) to 3 (‘I cannot do it’). The total score (range 0–33) reflected the degree of activity limitations, with higher scores indicating greater limitation. Responses of ‘do not do’ were treated as missing. Observations with > 20% missing items (*i.e*. > 2 of 11) were excluded from the total score calculation. The combined ADL/IADL scale demonstrated good internal consistency across waves (Cronbach's α: 0.85 in 2013, 0.86 in 2015, 0.90 in 2018) (Table S3 in the **Online Supplementary Document**). Exploratory factor analysis further indicated that the scale has good construct validity (Table S4 in the **Online Supplementary Document**).

#### Measurement of covariates

Potential confounders included socio-demographic factors (age, gender, retirement status, marital status, education level, pension receipt, health insurance) and lifestyle factors (smoking, alcohol use). Retirement status was dichotomised (retired / not retired). Marital status was categorised as living with or without a spouse. Education level was grouped as illiterate, primary school or below, junior high school, and high school or above. Pension, health insurance, smoking, and alcohol use were binary variables (yes/ no).

### Analytical strategy

Descriptive statistics were presented as mean ± standard deviation for continuous variables and count (%) for categorical variables. The functional form of the association between chronic diseases and falls was explored by first testing quadratic terms and plotting restricted cubic splines to assess nonlinearity. Following the absence of a significant nonlinear relationship (*P* for nonlinear > 0.05), a linear association was confirmed and visualised using a dose-response curve. We employed a mixed-effects logistic regression model with random intercepts for individuals and fixed effects for time to examine the longitudinal association between chronic diseases and falls. The basic model is specified as:



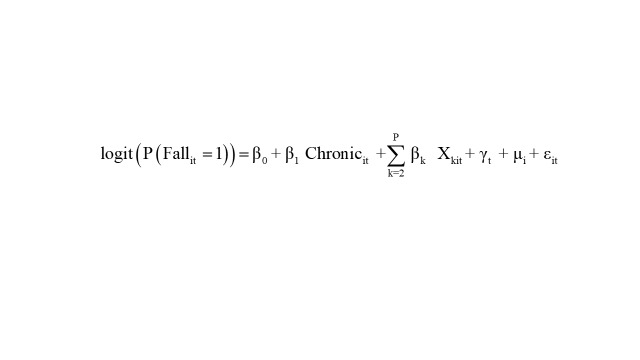



Where Fall_it_ is the binary fall outcome for individual ‘i’ at time ‘t’, β_0_ is the fixed intercept, Chronic_it_ is the number of chronic diseases for individual ‘i’ at time ‘t’, X_kit_ represents the covariate for individual ‘i’ at time ‘t’, γ_t_ are time-fixed effects for survey waves, μ_i_ is the individual-specific random intercept, ε_it_ is the residual error.

Then, Mediation analysis was conducted using a stepwise regression analysis method based on fully adjusted mixed-effect models, with bootstrapping (5000 repetitions) to test the significance of indirect effects. The proportion mediated was calculated as the ratio of the indirect effect to the total effect. Finally, we conducted four sensitivity analyses to test the robustness of our core findings. First, we re-fitted the main models using robust standard errors to improve the reliability of statistical inference. Second, we applied the more stringent two-way fixed effects model, incorporating both individual and time fixed effects. Third, we excluded variables that could potentially act as mediators (*i.e*. retirement status, pension, health insurance, smoking, and alcohol use) from the adjustment set to address concerns regarding over-adjustment. Finally, we excluded individuals who had experienced a fall in the prior wave to better isolate the temporal sequence and strengthen causal inference regarding the pathway from chronic diseases to falls through activity limitations.

All regression models were adjusted for age, gender, retirement status, marital status, education level, pension, health insurance, smoking, and alcohol use. Analyses were performed using Stata, version 15.1 (StataCorp LLC, College Station,Texas, USA), with a two-tailed *P*-value < 0.05 considered statistically significant.

## RESULTS

### Participants characteristics

Over the five-year follow-up, the cohort of 2400 participants demonstrated clear trends of accumulating health burdens. The prevalence of multimorbidity climbed markedly from 43.96 to 65.33% (**Table 1**). Concurrently, the mean score of activity limitations worsened from 1.47 to 2.47. Mirroring this progression, the incidence of falls rose steadily from 18.67 to 24.46%. The sample had a mean age of 61.2 years, was evenly distributed by gender, and predominantly had low educational attainment.

**Table 1:** Results of descriptive statistics

Variables	2013	2015	2018	Total
Fall, n (%)				
*No*	1952 (81.33)	1927 (80.29)	1813 (75.54)	5692 (79.06)
*Yes*	448 (18.67)	473 (19.71)	587 (24.46)	1508 (20.94)
Chronic diseases, n (%)				
*No chronic condition*	587 (24.46)	319 (13.29)	306 (12.75)	1212 (16.83)
*One chronic condition*	758 (31.58)	594 (24.75)	526 (21.92)	1878 (26.08)
*Multimorbidity*	1055 (43.96)	1487 (61.96)	1568(65.33)	4110 (57.08)
Chronic diseases number, x̄ ± SD	1.58 ± 1.42	2.15 ± 1.55	2.61 ± 2.01	2.11 ± 1.73
Activity limitations, x̄ ± SD	1.468 ± 3.43	1.798 ± 3.86	2.474 ± 4.88	1.913 ± 4.12
Age, x̄ ± SD	61.38 ± 9.66	60.12 ± 9.83	62.05 ± 10.13	61.18 ± 9.92
Gender, n (%)				
*Female*	1257 (52.44)	1245 (51.94)	1274 (53.08)	3776 (52.49)
*Male*	1140 (47.56)	1152 (48.06)	1126 (46.92)	3418 (47.51)
Retirement status, n (%)				
*Not retired*	2055 (94.79)	2099 (91.54)	1977 (97.05)	6131 (94.35)
*Retired*	113 (5.21)	194 (8.46)	60 (2.95)	367 (5.65)
Marital status, n (%)				
*No spouse or not cohabiting with spouse*	428 (17.87)	433 (18.07)	553 (23.04)	1414 (19.66)
*Cohabiting with spouse*	1967 (82.13)	1963 (81.93)	1847 (76.96)	5777 (80.34)
Education level, n (%)				
*Illiterate*	529 (26.77)	578 (27.79)	605 (25.21)	1712 (26.52)
*Primary education or below*	844 (42.71)	874 (42.02)	978 (40.75)	2696 (41.76)
*Junior high school*	387 (19.59)	408 (19.62)	527 (21.96)	1322 (20.48)
*High school or higher*	216 (10.93)	220 (10.58)	290 (12.08)	726 (11.25)
Pensions, n (%)				
*No*	500 (21.08)	69 (2.88)	267 (11.13)	836 (11.66)
*Yes*	1872 (78.92)	2329 (97.12)	2133 (88.88)	6334 (88.34)
Medical insurance, n (%)				
*No*	108 (4.53)	274 (11.42)	98 (4.09)	480 (6.68)
*Yes*	2274 (95.47)	2126 (88.58)	2301 (95.91)	6701 (93.32)
Smoking, n (%)				
*No*	1571 (85.10)	1745 (74.19)	1851 (77.13)	5167 (78.31)
*Yes*	275 (14.90)	607 (25.81)	549 (22.88)	1431 (21.69)
Alcohol use, n (%)				
*No*	1667 (69.63)	1663 (69.32)	1708 (71.17)	5038 (70.04)
*Yes*	727 (30.37)	736 (30.68)	692 (28.83)	2155 (29.96)
Number of groups	2400			

SD – standard deviation, x̄ – mean

### Correlation analysis

**Figure 2:**
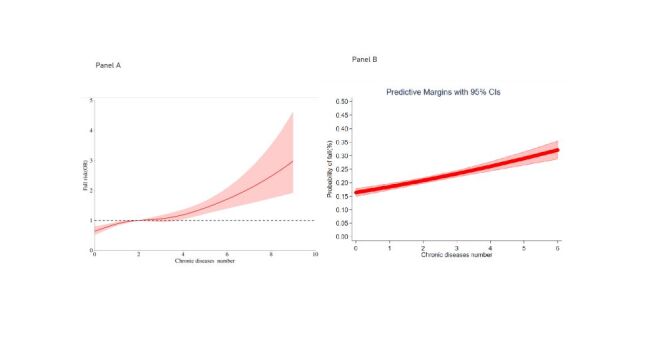
Association between number of chronic diseases and falls. **Panel A.** Restricted cubic spline of the association between chronic diseases and falls. **Panel B**. Dose-response relationship between chronic disease number and fall risk.

The quadratic term for the number of chronic diseases was not significant, and restricted cubic spline analysis confirmed no significant nonlinear relationship (**Figure 2**, Panel A; *P* for nonlinear > 0.05). Dose-response curves indicated a linear association between the number of chronic diseases and fall risk (**Figure 2**, Panel B).

### Mixed-effects logistic regression analysis

After adjusting for covariates and including time-fixed effects, the mixed-effects logistic regression model showed that having one chronic disease was significantly associated with an increased risk of falls (odds ratio (OR) = 1.340; 95% confidence interval (CI) = 1.062–1.691, *P* < 0.05) (**Table 2**), while multimorbidity was associated with even higher risk (OR = 1.741; 95% CI = 1.412–2.147, *P* < 0.01). Retirement status was associated with a lower fall risk (OR = 0.663; 95% CI = 0.473–0.930, *P* < 0.05), although this result should be interpreted cautiously due to the limited number of retired individuals.

**Table 2:** Results of mixed-effect logistic regression analyses*

Variables	OR	95% CI	*P*-value
Chronic diseases			
*No chronic condition*			
*One chronic condition*	1.340	1.062–1.691	0.014
*Multimorbidity*	1.741	1.412–2.147	<0.001
Age	1.003	0.996–1.011	0.336
Gender			
*Female*			
*Male*	0.957	0.832–1.100	0.533
Retirement status			
*Not retired*			
*Retired*	0.663	0.473–0.930	0.017
Marital status			
*No spouse or not cohabiting with spouse*			
*Cohabiting with spouse*	1.051	0.885–1.249	0.570
Education level			
*Illiterate*			
*Primary education or below*	0.980	0.825–1.164	0.817
*Junior high school*	1.108	0.902–1.361	0.326
*High school or higher*	1.193	0.933–1.525	0.159
Pensions			
*No*			
*Yes*	1.024	0.823–1.275	0.830
Medical insurance			
*No*			
*Yes*	0.934	0.714–1.222	0.619
Smoking			
*No*			
*Yes*	0.975	0.822–1.155	0.766
Alcohol use			
*No*			
*Yes*	0.973	0.832–1.138	0.730
Constant	0.136	0.071–0.259	<0.001
Year fixed effect		Yes	
Observations*		5221	
Number of groups		2334	

OR – odd ratio, CI – confidence interval

*The number of observations reflects person-wave data with complete information on all variables included in the regression models.

### Mediation analysis

The mediation analysis results strongly support our hypothesised pathway (**Table 3**; Tabel S4 in the **Online Supplementary Document**). In Model 1, each additional chronic disease was associated with a significant 0.138-unit increase in the log-odds of falling. Model 2 confirmed that a greater number of chronic diseases significantly predicted higher levels of activity limitations (β = 0.388, *P* < 0.01). When both the independent variable and the mediator were included in Model 3, the coefficient for chronic diseases attenuated from 0.138 to 0.108 but remained significant, indicating a partial mediation. The bootstrapping analysis yielded a significant indirect effect estimate of 0.029 with a 95% CI = 0.022–0.038, excluding zero. This confirms that the mediation effect via activity limitations was statistically substantial. The proportion mediated was calculated to be 21.08%, meaning that over one-fifth of the total effect of chronic disease count on fall risk operates through the pathway of worsening functional capacity.

**Table 3:** Results of mediating effects analyses*

	Model 1	Model 2	Model 3
**Variables**	**Fall**	**Activity limitations**	**Fall**
Chronic diseases number	0.138†	0.388†	0.108†
Activity limitations			0.075†
Control variables		Yes	
Year fixed effect		Yes	
Observations		5221	
Number of groups		2334	

*Adjusted for age, gender, retirement status, marital status, education level, pensions, medical insurance, smoking, and drinking.

†*P* < 0.01.

### Sensitivity analysis

In the sensitivity analyses, we first incorporated robust standard errors into the models, which yielded results showing no significant changes. Second, after applying the two-way fixed effects model, the results remained largely consistent with those from the primary analysis, despite a reduction in sample size. Third, the exclusion of potential mediating variables, including retirement status, pension receipt, health insurance, smoking, and alcohol use, also produced results that aligned closely with the main findings. Finally, after further excluding participants who had experienced falls in the prior wave, the analysis was limited to the 2015 and 2018 waves. Although some coefficients varied, the overall conclusions remained consistent with the primary analysis (Table S6 in the **Online Supplementary Document**).

## DISCUSSION

Our study revealed that the incidence of falls among Chinese middle-aged and older adults increased consistently from 18.67% at baseline to 24.46% in the 2018 wave. The overall fall incidence in this cohort was 20.94%, which aligns with previous estimates of 18–19% in similar populations [3]. Although this figure remains lower than the global average of 26.50% [19], the persistent upward trend warrants serious attention.

The results demonstrate that chronic diseases, particularly multimorbidity, significantly associate fall risk, consistent with previous cross-sectional studies. A linear dose-response relationship was observed between the number of chronic diseases and fall probability, suggesting cumulative or synergistic effects of multiple conditions [20]. The rising prevalence of multimorbidity, activity limitations, and falls underscores the importance of preventing new chronic conditions in this population.

Integrating mixed-effects modelling with longitudinal mediation analysis, this study examines the longitudinal relationships among chronic diseases, activity limitations, and falls using three waves of CHARLS data. The longitudinal data suggest that activity limitations mediate the association between chronic diseases and fall risk, underscoring its significance for fall prevention. Compared to cross-sectional or two-wave designs, our three-wave longitudinal approach and mixed-effects modelling provide stronger evidence for the temporal sequence and dynamic interrelationships among variables. This conclusion was strengthened by sensitivity analyses, including the use of robust standard errors and models that excluded potential mediators or prior fallers (to more stringently test temporal order), all of which yielded consistent results. The disablement process model proposes a pathway in which chronic diseases are linked to declined mobility via activity limitations, potentially raising the risk of disabling outcomes such as falls. This pathway is critical because only a subset of chronic conditions, such as arrhythmias that cause syncope or hypertension that leads to acute cerebral ischemia, are considered direct contributors to falls [21]. In contrast, the majority exert their influence indirectly through mediating mechanisms, including polypharmacy, pain, and, as this study highlights, limitations in activities of daily living. Our findings suggest that activity limitations explain roughly 21.08% of the association between chronic diseases and the risk of falls. This is consistent with previous research; for instance, a study by Li Huang et al. using both CHARLS and ELSA data sets reported that ADL impairment mediated over 20% of the effect of depressive symptoms on fall risk [22]. Similarly, an Irish prospective cohort study found that ADL limitations explained 10.7% of the association between multimorbidity and falls [23]. The slightly lower proportion in the latter may be attributable to differences in cultural context, data types, or analytical methods. Nonetheless, the mediating role of activity limitations remains considerable across studies. Existing evidence indicates that the number of chronic conditions in older adults affects both ADL and IADL, with the cumulative burden of comorbidities directly leading to functional decline and even disability [15]. This occurs as multimorbidity can trigger interactions between different diseases, thereby compromising compensatory mechanisms. Moreover, older adults with multiple chronic conditions often require complex medication regimens, and polypharmacy increases the risk of adverse drug interactions [24], which may further exacerbate activity limitations. It is also well-established that mobility limitations are not only a risk factor for falls but can also be a consequence of fall incidents [25,26]. Falls among middle-aged and older adults often occur during routine daily activities. Activity limitations can impair gait, balance, and lower-limb strength by compromising musculoskeletal and other physiological functions, thereby elevating fall risk [27]. Notably, the disablement process model posits that the end stage of a deteriorating disability trajectory is the loss of independence in daily activities – a severe outcome that falls can directly accelerate. Therefore, timely intervention to mitigate or improve activity limitations may also help prevent this adverse progression.

The pathological damage resulting from the progression of chronic diseases is often irreversible, implying that their influence on fall risk is likely persistent. Although the underlying pathology may not be reversible, the symptoms and clinical manifestations of chronic conditions can be significantly improved through targeted interventions [28,29]. Crucially, the level of activity limitations can be controlled, delayed, or even reversed through specific measures, such as rehabilitation training focused on functional recovery and regular physical exercise. Our findings, which identify activity limitations as a key factor in the pathway linking chronic diseases to falls, suggest that mitigating functional decline may be a promising target for risk reduction. Consistent with this perspective, multiple randomised controlled trials have demonstrated that physical exercise is effective in preventing major mobility disability [30–32] and may also confer cognitive benefits [33]. Therefore, in clinical practice, assessing activity limitations can serve as a valuable strategy for identifying individuals at higher fall risk, and evidence-based interventions like structured exercise and balance training should be considered for those with or at risk of functional decline. Future intervention studies specifically targeting ADL/IADL limitations in multimorbid populations are needed to directly test the causal impact on fall prevention.

This study has the following limitations. First, measuring chronic diseases by number rather than type or severity may mask heterogeneity in disease combinations. Second, the study relied on self-reported data for both chronic disease diagnoses and fall history, which may introduce underreporting and recall bias, particularly among older adults. If such measurement errors are non-differential, they would likely bias our effect estimates toward the null, rendering the observed associations conservative. Third, the observational design cannot fully rule out residual confounding (*e.g*. from unmeasured or time-varying factors), reverse causality, or bias due to measurement error. Finally, the generalisability of our findings is subject to two main considerations. First, the restriction to participants with complete three-wave data may favour a relatively healthier subset. Second, the sample is drawn from a single national context. Multinational replications and strategies to retain frailer participants are needed.

## CONCLUSIONS

The proportion of middle-aged and older adults with multimorbidity is increasing in China, accompanied by a linear rise in fall risk. Activity limitations mediate a significant portion (21.08%) of the longitudinal association between chronic diseases and falls. Preventing new chronic conditions and implementing fall risk assessments that include functional measures are urgent public health priorities. For those already living with multimorbidity, interventions such as physical exercise and rehabilitation programmes may be prioritised as a strategy to mitigate fall risk.

## Additional material


Online Supplementary Document
