# Supplementary material for: Longitudinal association between chronic diseases and fall risk among middle-aged and older adults in China: the mediating role of activity limitations
Source: J Glob Health. 2026 Mar 13;16:04106. doi: 10.7189/jogh.16.04106 (PMC12983047; doi:10.7189/jogh.16.04106)
Supplement: Online Supplementary Document [file jogh-16-04106-s001.pdf]

Supplement to: Huang D, Fu Y, Yang W, Xu B, Yang Z. Longitudinal association between chronic diseases and fall risk among middle-aged and older adults in China: the mediating role of activity limitations. J Glob Health. 2026;16:04106.

Table S1. Outline of JoGH's Guidelines for Reporting Analyses of Big Data Repositories Open to the Public (GRABDROP) items.

JoGH guideline item	Author's Response
1. Please list all papers published by each co-author in previous 3 years that were based on secondary analysis of a big data repository	1. Zhao R, Wang J, Lou J, Liu M, Deng J, **Huang D**, Fang H. The effect of education level on depressive symptoms in Chinese older adults–parallel mediating effects of economic security level and subjective memory ability. BMC Geriatr 24, 635 (2024).<br>2. Lou J, Wang J, **Fu Y**, **Huang D**, Liu M, Zhao R, Deng J. Association between Oral Health and Depressive Symptoms in Chinese Older Adults: The Mediating Role of Dietary Diversity. Nutrients. 2024; 16(8):1231.<br>3. **Huang D**, Wang J, Fang H, **Fu Y**, Lou J. Longitudinal association of chronic diseases with depressive symptoms in middle-aged and older adults in China: mediation by functional limitations, social interaction, and life satisfaction. J Glob Health. Sep 29, 2023;13:04119.
2. Please explain the key elements of your study design and the use of the available datasets that make your study an original scientific contribution	This study provides an original contribution by offering the first longitudinal, empirical alignment with the theoretical framework of the disablement process model in a Chinese middle-aged and older population. Through concurrent examination of chronic diseases, functional limitations, and falls across three waves of CHARLS data using an integrated mixed-effects and longitudinal mediation approach, we observed a consistent gradient of risk between the number of chronic conditions and fall probability. This analysis further indicated that differences in functional status could help explain over one-fifth of this observed risk gradient, thereby extending the longitudinal evidence beyond prior cross-sectional findings.
3. Please list all publications that addressed similar research questions in the same dataset and indicate where you cited them in your paper	Tang SL, Liu MX, Yang TL, Ye CY, Gong Y, Yao L, Xu Y, Bai YM. Association between falls in elderly and the number of chronic diseases and health-related behaviors based on CHARLS 2018: health status as a mediating variable. BMC Geriatr 2022, 22(1):13, cited as reference 11 in tihs paper.<br>Tian Y, Zhou XZ, Jiang Y, Pan YD, Liu XFD, Gu XB. Bidirectional association between falls and multimorbidity in middle-aged and elderly Chinese adults: a national longitudinal study. Scientific Reports 2024, 14(1), cited as reference 12 in tihs paper.

4. Please explain how you addressed multiple testing through an appropriately rigorous statistical threshold and indicate this in the methods section	To address multiple testing, we pre-specified our primary inferences to the core exposure-mediator-outcome pathway. The significance of the key mediation effect was assessed using bias-corrected bootstrap confidence intervals (5,000 repetitions), a robust method that does not rely on normality assumptions. For other comparisons, we emphasized the magnitude of effect sizes (e.g., standardized mean differences) alongside p-values, preventing an over-reliance on statistical significance from numerous tests.
5. Please declare to what extent have AI chatbots been used in developing your paper and to which parts of the paper did they contribute	We declare that AI chatbots were used solely for language editing.

Adapted from: Rudan I, Song P, Adeloye D, Campbell H. Journal of Global Health's Guidelines for Reporting Analyses of Big Dat a Repositories Open to the Public (GRABDROP): preventing 'paper mills', duplicate publications, misuse of statistical inference, and inappropriate use of artificial intelligence. J Glob Health. 2025;15:01004.

Table S2. Distribution of chronic disease counts across waves

Number of Chronic Diseases	2013, N (%)	2015, N (%)	2018, n (%)	Total Person-Waves, N (%)
0	587 (24.46)	319 (13.29)	306 (12.75)	1212 (16.83)
1	758 (31.58)	594 (25.75)	526 (21.92)	1878 (26.08)
2	519 (21.63)	636 (26.50)	481 (20.04)	1636 (22.72)
3	287 (11.96)	399 (16.63)	404 (16.83)	1090 (15.14)
4	150 (6.25)	273 (11.38)	266 (11.08)	689 (9.57)
5	65 (2.71)	110 (4.58)	189 (7.88)	364 (5.06)
6	22 (0.92)	43 (1.79)	122 (5.08)	187 (2.60)
7	10 (0.42)	19 (0.79)	60 (2.50)	89 (1.24)
8	2 (0.08)	6 (0.25)	28 (1.17)	36 (0.50)
9	0 (0.00)	1 (0.04)	8 (0.33)	9 (0.13)
10	0 (0.00)	0 (0.00)	5 (0.21)	5 (0.07)
11	0 (0.00)	0 (0.00)	3 (0.13)	3 (0.04)
12	0 (0.00)	0 (0.00)	1 (0.04)	1 (0.01)
13	0 (0.00)	0 (0.00)	1 (0.04)	1 (0.01)

N – number, % – percentage

Table S3. Results of internal consistency reliability analysis for the activity limitations scale

Scale	Items	Cronbach's coefficient	Range of item-total correlation
Full sample			
Total scale	11	0.8760	0.4873–0.7941
ADL subscale	6	0.8181	0.4873–0.7394
IADL subscale	5	0.8019	0.5624–0.7941
2013			
Total scale	11	0.8460	0.3586–0.7662
ADL subscale	6	0.7587	0.3586–0.7140
IADL subscale	5	0.7703	0.5005–0.7662
2015			
Total scale	11	0.8631	0.4614–0.8005
ADL subscale	6	0.8074	0.5001–0.7292
IADL subscale	5	0.7675	0.4614–0.8005
2018			
Total scale	11	0.8963	0.5530–0.8029
ADL subscale	6	0.8532	0.5530–0.7581
IADL subscale	5	0.8321	0.6501–0.8029

Tabel S4. Results of exploratory factor analysis for the activity limitations scale (varimax rotation)*

Item	Full Sample			2013			2015			2018		
	**Factor 1 (ADL)**	**Factor 2 (IADL)**	**Communality (h^2)**	**Factor 1 (ADL)**	**Factor 2 (IADL)**	**Communality (h^2)**	**Factor 1 (ADL)**	**Factor 2 (IADL)**	**Communality (h^2)**	**Factor 1 (ADL)**	**Factor 2 (IADL)**	**Communality (h^2)**
ADL1	**0.755**	0.2623	0.3598	**0.7048**	0.2983	0.4143	**0.7634**	0.1463	0.3959	**0.7853**	0.2766	0.3067
ADL2	**0.6402**	**0.4324**	0.4032	**0.6021**	**0.4583**	0.4274	**0.7461**	0.2354	0.3879	**0.5853**	**0.5121**	0.3953
ADL3	**0.6411**	0.2514	0.5257	**0.5598**	0.2655	0.6161	**0.6050**	0.2338	0.5793	**0.7276**	0.2268	0.4192
ADL4	**0.7831**	0.1999	0.3468	**0.7372**	0.2054	0.4143	**0.7895**	0.0738	0.3713	**0.7922**	0.2475	0.3112
ADL5	**0.6767**	0.1807	0.5095	**0.6909**	0.1568	0.4981	**0.6482**	0.1088	0.5680	**0.6792**	0.2536	0.4744
ADL6	**0.5537**	0.1411	0.6735	**0.4453**	0.0295	0.8008	**0.5278**	0.1277	0.7052	**0.6339**	0.1881	0.5628
IADL1	**0.4525**	**0.6402**	0.3853	0.3828	**0.6515**	0.4290	**0.6210**	**0.4703**	0.3932	0.3903	**0.7093**	0.3447
IADL2	**0.4134**	**0.6917**	0.3507	0.3293	**0.7255**	0.3652	**0.6151**	**0.4992**	0.3725	0.3466	**0.7538**	0.3116
IADL3	0.2540	**0.7616**	0.3554	0.1776	**0.8021**	0.3251	**0.4346**	**0.6111**	0.4377	0.2250	**0.7830**	0.3363
IADL4	0.0348	**0.7848**	0.3828	0.0516	**0.7299**	0.4645	0.0661	**0.8051**	0.3475	0.0757	**0.7681**	0.4043
IADL5	0.2569	**0.5542**	0.6268	0.2921	**0.4270**	0.7323	0.1111	**0.6539**	0.5600	0.3643	**0.5643**	0.5488
KMO		0.9096			0.8834			0.8992			0.9181	
Bartlett's χ^2		30586.569 (p <0.001)			8288.420 (p <0.001)			9503.359 (p <0.001)			12225.327 (p <0.001)	

*Factor loadings > 0.4 are displayed in bold.

Table S5. Results of bootstrapping analyses*

Effect	Estimate	Boot SE	Boot [95% CI]	
			Lower	Upper
Indirect effect（X→M→Y）	0.029	0.004	0.022	0.038
Direct effect（X→Y）	0.108	0.021	0.067	0.149
Total effects	0.138	0.021	0.096	0.176

CI – confidence interval

*Number of bootstrap samples for bias-corrected bootstrap confidence intervals: 5,000.

Table S6. Results of Sensitivity Analyses*

Variables	Model 1	Model 2	Model 3	Model 4	Model 5	Model 6	Model 7	Model 8	Model 9	Model 10	Model 11	Model 12
	Fall	Activity limitations	Fall	Fall	Activity limitations	Fall	Fall	Activity limitations	Fall	Fall	Activity limitations	Fall
Chronic diseases number	0.138†	0.388†	0.108†	0.117†	0.444†	0.086†	0.126†	0.416†	0.095†	0.121†	0.363†	0.095†
activity limitations			0.075†			0.067†			0.071†			0.069†
Control variables	Yes	Yes	Yes	Yes	Yes	Yes	Yes	Yes	Yes	Yes	Yes	Yes
Year fixed effect	Yes	Yes	Yes	Yes	Yes	Yes	Yes	Yes	Yes	Yes	Yes	Yes
Individual fixed effect	No	No	No	Yes	Yes	Yes	No	No	No	No	No	No
Observations	5221	5221	5221	2065	5221	2,065	6326	6326	6326	3143	3143	3143
Number of groups	2334	2334	2334	782	2334	782	2394	2394	2394	2110	2110	2110

*Models 1–3 incorporated robust standard errors; Models 4–6 applied the two-way fixed effects model; Models 7–9 excluded potential mediating variables including retirement, smoking, alcohol use, pension, and health insurance; Models 10–12 removed samples that reported falls in the prior wave. The number of observations reflects person-wave data with complete information on all variables included in the regression models.

†$P < 0.01$.
